# Carbon dioxide receptor genes and their expression profile in *Diabrotica virgifera virgifera*

**DOI:** 10.1186/s13104-015-1794-4

**Published:** 2016-01-08

**Authors:** Thais B. Rodrigues, Etsuko N. Moriyama, Hang Wang, Chitvan Khajuria, Blair D. Siegfried

**Affiliations:** CAPES Foundation, Ministry of Education of Brazil, Brasília, DF 70040-020 Brazil; Federal University of Lavras, Lavras, Minas Gerais Brazil; School of Biological Sciences and Center for Plant Science Innovation, University of Nebraska-Lincoln, Lincoln, NE 68583 USA; Department of Entomology, University of Nebraska-Lincoln, Lincoln, NE 68583 USA

**Keywords:** *D. v. virgifera*, Western corn rootworm, CO_2_ receptors, Gustatory receptors, qRT-PCR, Phylogenetic analyses, Gr

## Abstract

**Background:**

*Diabrotica virgifera virgifera*, western corn rootworm, is one of the most devastating species in North America. *D. v. virgifera* neonates crawl through the soil to locate the roots on which they feed. Carbon dioxide (CO_2_) is one of the important volatile cues that attract *D. v. virgifera* larvae to roots.

**Results:**

In this study, we identified three putative *D. v. virgifera* gustatory receptor genes (*Dvv_Gr1*, *Dvv_Gr2*, and *Dvv_Gr3*). Phylogenetic analyses confirmed their orthologous relationships with known insect CO_2_ receptor genes from *Drosophila*, mosquitoes, and *Tribolium*. The phylogenetic reconstruction of insect CO_2_ receptor proteins and the gene expression profiles were analyzed. Quantitative analysis of gene expression indicated that the patterns of expression of these three candidate genes vary among larval tissues (i.e., head, integument, fat body, and midgut) and different development stages (i.e., egg, three larval stages, adult male and female).

**Conclusion:**

The *Dvv_Gr2* gene exhibited highest expression in heads and neonates, suggesting its importance in allowing neonate larvae to orient to its host plant. Similar expression patterns across tissues and developmental stages for *Dvv_Gr1* and *Dvv_Gr3* suggest a potentially different role. Findings from this study will allow further exploration of the functional role of specific CO_2_ receptor proteins in *D. v. virgifera*.

## Background

Many insects are able to detect carbon dioxide (CO_2_) in the environment for a variety of purposes, such as the location of their vertebrate hosts by hematophagous insects [1] evaluation of floral quality by lepidopterans [2], and the regulation of potentially lethal CO_2_ concentrations by social insects in colonies [3]. Insect herbivores, such as the western corn rootworm *Diabrotica virgifera virgifera* LeConte (Coleoptera: Chrysomelidae), use CO_2_ as an important host finding cue [4].

*Diabrotica virgifera virgifera* is one of the most devastating corn pests in North America [5]. The common name, western corn rootworm, refers to the larval life stage that feeds on corn roots, which moves through the soil to find roots of a suitable host [6]. Neonates that hatch in the spring from overwintering eggs must crawl through the soil to locate the roots on which they feed. It has been suggested that CO_2_ emitted by corn roots is one of the important volatile cues that attract *D. v. virgifera* larvae to corn roots [4].

In general, a chemical signal from the environment is converted to an electrical signal that can be interpreted by the insect nervous system due the binding of a ligand to a receptor protein [7]. Most of these chemosensory proteins are recognized as members of two evolutionarily related chemosensory receptor families; the odorant receptors (ORs) and gustatory receptors (GRs) [8–10]. Three groups of GR receptors (GR1–3) appear to contribute to the detection of CO_2_ in insects [11]. In *Drosophila melanogaster*, *DmGR21a* and *DmGR63a* (belonging to the Gr1 and Gr3 groups, respectively) are co-expressed in olfactory receptor neurons of the sensilla on the antennae that are sensitive to CO_2_ and both proteins are required for CO_2_ detection [12–14]. In mosquitos and other insects, a third group of *Gr* genes is also identified and designated as *Gr2* [11]. Although all three *Gr* genes are expressed in the sensilla located on the maxillary palps in mosquitoes, expression of only *Gr1* and *Gr3* is required for CO_2_ perception [13, 15, 16]. The orthologs of three CO_2_ receptor genes have been identified from a lepidopteran species (the silk moth *Bombyx mori*) and from several coleopteran species (the red flour beetle *Tribolium castaneum*, the mountain pine beetle *Dendroctonus ponderosae*, and the European spruce bark beetle *Ips typographus*) [11, 17]. Interestingly, in *D. ponderosae*, the *Gr2* gene was only identified from the draft genome and from larval RNAseq data, but not from antennal transcriptomes [17, 18].

In this study, we have taken significant steps to further investigate CO_2_ receptor genes in *D. v. virgifera*. We identified three putative CO_2_ receptor genes from a larval *D. v. virgifera* transcriptome [19] and we characterized the expression of those genes in different *D. v. virgifera* tissues and developmental stages.

## Methods

### Identification of CO_2_ receptor genes from the D. v. virgifera transcriptomes

Protein sequences of the following CO_2_ receptor genes were obtained from the National Center for Biotechnology Information database: DmGr21a (NM_078724.6) and DmGr63a (NM_001144411.1) from *D. melanogaster*, GPRGR24 (DQ989013.1), GPRGR22 (DQ989011.1), and GPRGR23 (XM_312786.3) from *Anopheles gambiae*, and TcGr1 (AM292331.2), TcGr2 (XM_008193301.1), and TcGr3 (XM_001814609.2) from *T. castaneum*. These protein sequences were used as the queries for tblastn similarity searches [20] against the combined transcriptome obtained from *D. v. virgifera* eggs, neonates, and midgut of 3rd instar larvae [19] with 1 × 10^−100^ as the E-value threshold to identify CO_2_ receptor gene candidates in *D. v. virgifera*.

In order to confirm our assembled CO_2_ receptor transcript sequences and examine their exon–intron structures, we also compared the protein sequences of the three CO_2_ receptor candidates we obtained against the draft *D. v. virgifera* genome sequences (Hugh M. Robertson, personal communication) using tblastn similarity search. Prediction of membrane protein topology was achieved using TOPCONS [21].

### Phylogenetic reconstruction of insect CO_2_ receptor proteins

Multiple alignments of CO_2_ receptor protein sequences were generated using MAFFT (ver. 7.215) with the L-INS-i algorithm [22]. The maximum-likelihood phylogenetic tree was reconstructed using PhyML (ver. 3.0) [23] with the LG substitution model. Non-parametric bootstrap analysis was performed with 1000 pseudoreplicates [24].

### Expression studies of the three Dvv_Gr genes

#### Insect

The adults and eggs of a non-diapause strain of *D. v. virgifera* used in this study were purchased from Crop Characteristics (Farmington, MN). The adults were held in rearing cages with artificial diet and maintained in a growth chamber with 23 ± 1 °C and 75 ± 5 % relative humidity. The freshly laid eggs received in petri dish were wrapped with foil and kept in an incubator at 27 ± 1 °C and 75 ± 5 % relative humidity until hatching.

#### Sample collection

The gene expression profiles of the three putative CO_2_ receptors genes were analyzed in two different experiments involving four different tissues and six developmental stages. Five 3rd instars were dissected for samples from integument, midgut, fat body and head with thorax. The same tissues from five 3rd instar larvae were pooled as a single replicate. All collected tissues and whole bodies from different development stages were snap-frozen in liquid nitrogen and stored at −80 °C until used. The samples for different development stages included pooled samples of eggs, 1st (30 larvae), 2nd (15 larvae) and 3rd (6 larvae) instar, and individual female and male adults. Each treatment condition was replicated three times.

#### RNA extraction and cDNA synthesis

Total RNA was extracted using RNeasy Mini Kit (Qiagen) according to the manufacture’s instructions. The RNA integrity was confirmed on 1 % agarose electrophoresis gels and NanoDrop-1000 (Thermo) before cDNA synthesis. RNA (1000 ng) from each sample was used to synthesize the cDNA using the QuantiTect Reverse Transcription kit (QIAGEN) according to manufacturer’s instructions. The cDNAs were quantified using a NanoDrop-1000 and stored in −20 °C until used.

#### Primer design and efficiency test

Based on the nucleotide sequences of the three *Dvv_Gr* genes, as well as two reference genes, EF1a (elongation factor 1a) and beta-actin [25], the primers for qPCR were designed using Primer3Plus (http://www.bioinformatics.nl/cgi-bin/primer3plus/primer3plus.cgi/). The primer efficiency test (E) and correlation coefficients (R^2^) were calculated and qRT-PCR assays were performed using Fast SYBR Green Master Mix (Applied Biosystems, Cat. 4385612) on Applied Biosystems^®^ 7500 Real-Time PCR Systems at default setting (Table 1).

**Table 1:** General information of the primers for qPCR analyzes

Gene name	Primer Sequence (5′–3′)	Amplicon (bp)	E (%)	R^2^
*Dvv_Gr1*	Forward: GTGGCACAGCATTGCTTA	221	94	0.970
	Reverse: CTATACGCCCTGCCCAAC			
*Dvv_Gr2*	Forward: GAACTAAGCGAGCTCCTCCA	192	108.8	0.989
	Reverse: CAGAAGCACCATGCAATACG			
*Dvv_Gr3*	Forward: CTGGATGAATGACCATGCAC	184	104.6	0.992
	Reverse: ATCCTCGGGGATGCTTATCT			

*E* amplification efficiency, *R* ^*2*^ correlation coefficients

#### Real-time quantitative PCR (qRT-PCR) and data analysis

The qPCR experiments were conducted with SYBR Green PCR Master Mix kit following the manufacturer’s instructions. Briefly, the PCR mixture contained 1 µL synthesized cDNA (~35 ng), 0.2 µL of each primer (10 µM), 5 µL of the SYBR green PCR master mix and 3.6 µL of ddH_2_O. All reactions were carried out in triplicate per template in a final volume of 10 µL. qRT-PCR reactions were performed on the 7500 Fast Real-Time PCR system (Applied Biosystems) with the following cycling conditions: one cycle at 95 °C (20 s), followed by 40 cycles of denaturation at 95 °C (3 s), annealing and extension at 60 °C for 30 s. At the end of each qRT-PCR reaction, a melting curve was generated to confirm a single peak and rule out the possibility of primer-dimer and non-specific product formation. The EF1a (elongation factor 1a) and actin genes were used as endogenous controls for tissue and stage experiments, respectively [25]. Third instar larvae were selected as reference stage for comparisons in both experiments.

The 2^−ΔΔCt^ method [26] was used to calculate the relative expression level of target gene in the samples as compared to control sample. The one-way analysis of variance (ANOVA) was used for statistical analysis and Tukey test (at *P* < 0.05) for statistical significance with Sigma Plot Program (version 12.0).

## Results

### Identification of D. v. virgifera CO_2_ receptor genes

**Fig. 1:**
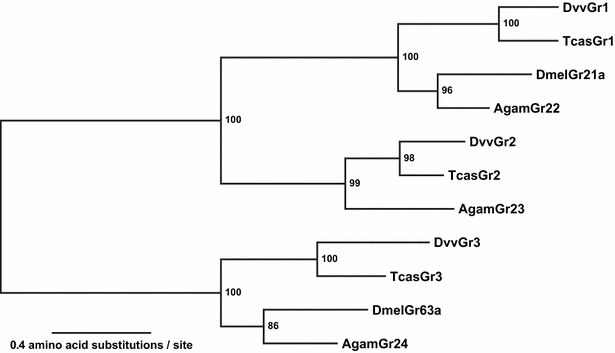
The maximum-likelihood phylogeny of insect CO_2_ receptor proteins. Three CO_2_ receptor proteins identified from *D. v. virgifera* (Dvv) were compared against orthologous proteins from *T. castaneum* (Tcas), *D. melanogaster* (Dmel), and *A. gambiae* (Agam). For the *T. castaneum* proteins, the naming convention proposed by Robertson and Kent [11] is used. The phylogeny is the consensus tree based on bootstrap analysis with 1000 pseudoreplicates. The *numbers* at nodes show the bootstrap supporting values (%)

Three CO_2_ receptor gene candidates were identified from the combined transcriptome assembled from egg, neonates, and midgut of 3rd instar larvae from *D. v. virgifera* [19]. The maximum-likelihood phylogenetic analysis at the amino-acid level including CO_2_ receptors from *D. melanogaster*, *A. gambiae*, and *T. castaneum* showed clear orthologous relationships for the three candidate genes (Fig. 1). We therefore named these *D. v. virgifera* genes as *Dvv_Gr1*, *Dvv_Gr2*, and *Dvv_Gr3* following the convention proposed by Robertson and Kent [11]. All three CO_2_ receptor proteins were predicted to have seven transmembrane regions with intercellular N-terminals. This topology, which is opposite to those of the regular 7-transmembrane G-protein coupled receptors, is consistent with what has been reported for other insect chemoreceptors [27].

### Structures of D. v. virgifera CO_2_ receptor genes

**Fig. 2:**
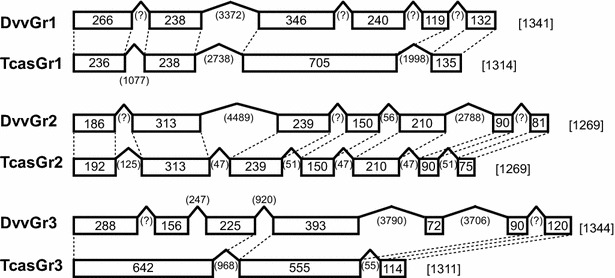
Comparison of CO_2_ receptor gene structures between *D. v. virgifera* and *T. castaneum*. Exons and introns located within the coding regions are depicted by boxes and peaks with their lengths (bp), respectively. The total length (bp) of each coding region is shown in *square bracket*s. *D. v. virgifera* gene structures were determined by comparing transcript sequences and the draft genome sequence (Hugh Robertson, personal communication). Exon–intron structures for *T. cantaneum* CO_2_ receptor genes are based on the annotations available in the BeetleBase [39]: TC030102 (TcasGr1), TC030103 (TcasGr2), and TC030104 (TcasGr3)

We confirmed the assembled sequences of the three CO_2_ receptor transcripts against the draft *D. v. virgifera* genome sequences (Hugh M. Robertson, personal communication). This comparison also enabled us to identify intron–exon structures of each CO_2_ receptor gene. While the *Dvv_Gr2* gene structure is consistent with the *Tribolium* ortholog (*TcGr2*), the other two *Dvv_Gr* genes have more introns compared to their *Tribolium* orthologs (Fig. 2).

### Expression studies of the three Dvv_Gr genes

**Fig. 3:**
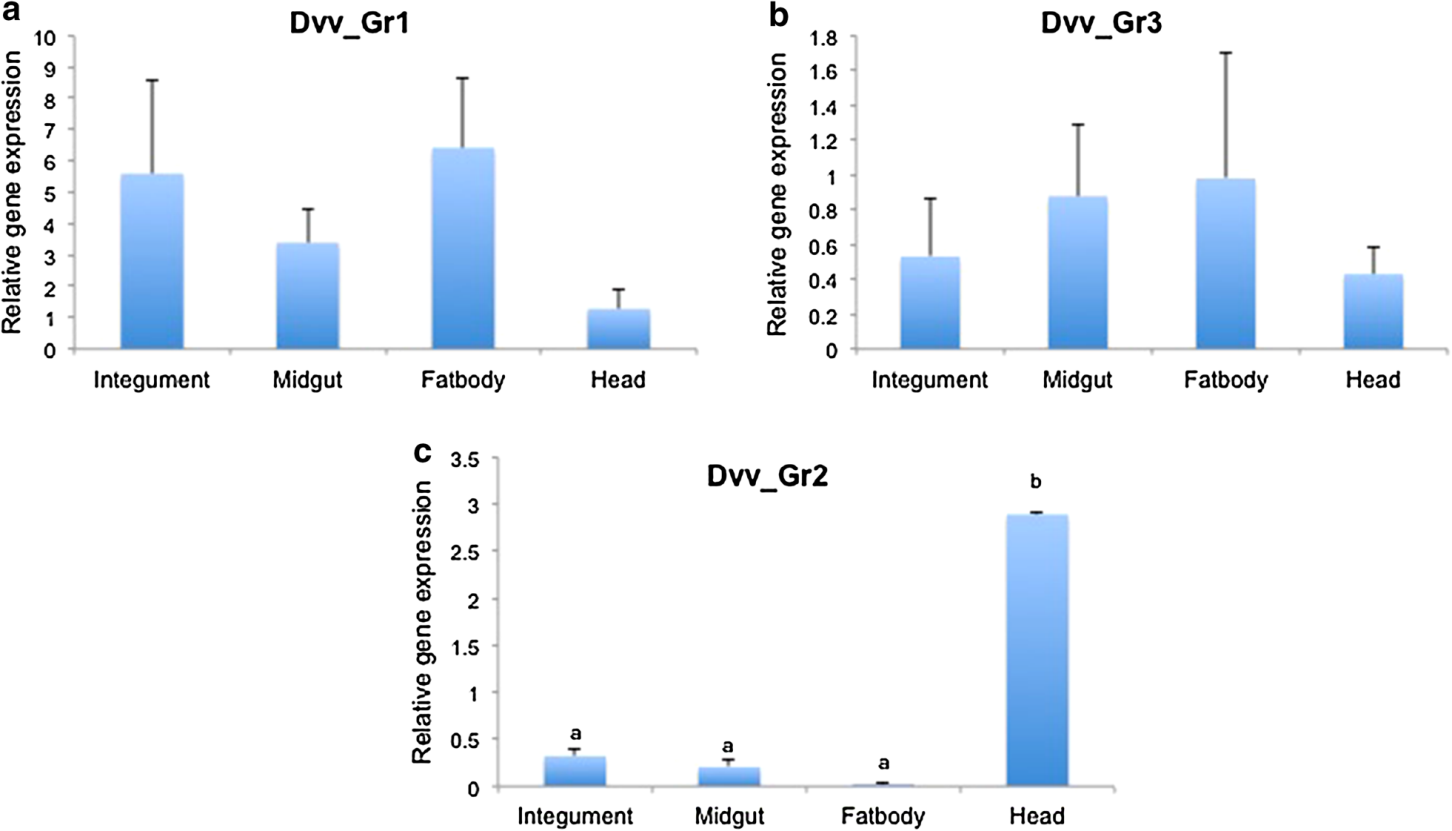
Expression of CO_2_ receptors (**a** Dvv_Gr1, **b** Dvv_Gr3, **c** Dvv_Gr2) in different tissues of *Diabrotica v. virgifera*. For qRT-PCR, relative expression of Dvv_Gr genes in different tissues was measured and normalized to an endogenous control (EF1a) as described in the “Methods” section. *Values* represent the means and the standard deviation of three analytical replicates on samples that contain tissue from five 3rd instar larvae. *Different letters* above the *bars* reflect significantly different expression levels (ANOVA of Tukey Test, *P* < 0.050)

The expression levels of three *D. v. virgifera* CO_2_ receptor gene candidates were quantified and compared among different tissues and development stages. No significant difference was observed in expression levels of *Dvv_Gr1* and *Dvv_G3* among different tissues including larval integument, midgut, fatbody and head (Fig. 3a, b). In contrast, the expression of *Dvv_Gr2* varied significantly among different tissues and was expressed almost five-fold higher in the head as compared to expression in integument and midgut (Fig. 3c).

**Fig. 4:**
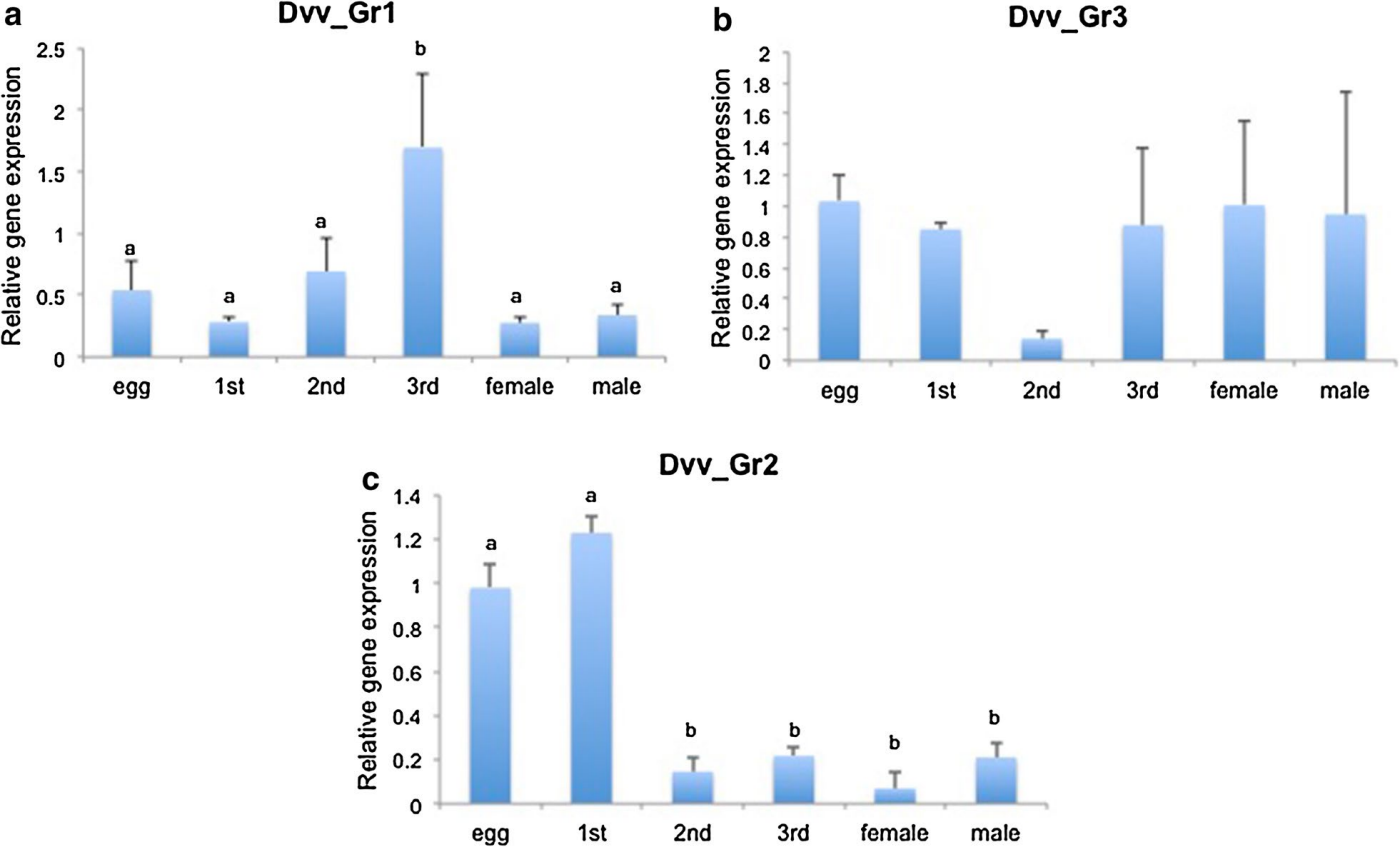
Expression of CO_2_ receptors (**a** Dvv_Gr1, **b** Dvv_Gr3, **c** Dvv_Gr2) in different development stages of *Diabrotica v. virgifera*. For qRT-PCR, relative expression of Dvv_Gr genes in different stages was measured and normalized to an endogenous control (actin) as described in the “Methods” section. *Values* represent the means and the standard deviation of three analytical replicates on samples that contain tissue from five 3rd instar larvae. *Different letters* above the *bars* reflect significantly different expression levels (ANOVA of Tukey Test, *P* < 0.050)

No significant differences in expression of *Dvv_Gr1* among different developmental stages were observed although third instars appeared to exhibit higher expression compared to second instars (Fig. 4a). *Dvv_Gr3* did not vary in expression level among the developmental stages analyzed (Fig. 4b). In contrast, the level of expression of *Dvv_Gr2* gene in eggs and first instar larvae was significantly (fivefold) higher, compared to the other instars and adults (Fig. 4c).

## Discussion

Many studies have been conducted to identify and validate the function of chemosensory receptors in insects and their role in allowing insects to perceive their environment [11, 13, 16, 17, 28–31]. The three genes (*Dvv_Gr1*, *Dvv_Gr2*, and *Dvv_Gr3*) were identified from a *D. v. virgifera* transcriptome based on significant similarity to CO_2_ receptor genes from *D. melanogaster*, *A. gambiae*, and *T. castaneum* and confirm their existence in western corn rootworms. Importantly, the relative expression of these genes among different larval tissues and developmental stages suggest a possible role for at least one of these genes in orientation to CO_2_ and potentially host finding. The entire larval stage of *D. v. virgifera* is spent underground feeding on roots. Neonates must crawl relatively long distances through the soil to locate roots of a suitable host after hatching [5]. Previous research has shown that neonates are attracted to carbon dioxide in the soil, which may serve as a mechanism of host finding [6]. *Gr1* and *Gr3* orthologs in *Drosophila* (*Dm21a* and *Dm63a*) were found to mediate carbon dioxide detection in adults [12–14]. However, for the three CO_2_ receptor orthologs identified from the *T. castaneum* genome (*TcasGr1*, *TcasGr2*, and *TcasGr3*) [32], their function has yet to be documented. No expression differences were observed among the four different *D. v. virgifera* tissues for *Dvv_Gr1* and *Dvv_Gr3* genes (Fig. 3a, b). However, *Dvv_Gr2* was highly expressed in the head as compared to fat body, integument, and midgut (Fig. 3c). The higher expression of *Dvv_Gr2* in the head may suggest localization of the receptor to chemosensory organs associated with mouthparts and a specific role for this gene as a carbon dioxide receptor in *D. v. virgifera* larvae. Similar expression patterns from the two CO_2_ receptor genes from *Drosophila* where expression is localized in olfactory receptor neurons of the sensilla on the antennae have been previously noted [13]. Similarly, all three CO_2_ receptor genes in mosquitoes are expressed on the maxillary palps [13, 15, 16].

Erdelyan et al. [16] reported that in *Aedes aegypti* and *Culex pipiens quinquefasciatus,* the *Gr1* and *Gr3* genes were expressed at higher levels in adults than in larvae and pupae. For blood-feeding mosquitoes, CO_2_ is a chemical stimulus emitted in the breath of animal hosts and produces host-seeking behaviors in adult mosquitos [33, 34]. In contrast, CO_2_ is used by *D. v. virgifera* larvae to locate the roots of growing corn plants for feeding [6, 35]. Therefore, the relatively high expression of *Dvv_Gr2* gene in the head might indicate a possible role for this gustatory receptor gene that mediates CO_2_ detection in *D. v. virgifera* larvae.

The level of expression of the *Dvv_Gr2* gene in eggs and first instar larvae was higher than in other development stages (Fig. 4c). CO_2_ is given off by growing corn roots in the soil or potentially other sources of CO_2_ that are associated with plant growth, and neonate larvae that hatch in the spring from overwintering eggs must crawl through the soil to locate the roots on which they feed [6]. Higher expression of *Dvv_Gr2* gene in eggs and first instars is consistent with a possible role in host finding, which is different from mosquitoes that need to orient to hosts in the adult stage [16]. Interestingly, for *D. ponderosae*, the two *Gr* genes (*Gr1* and *Gr3*) were identified from an antenna-specific transcriptome but *Gr2* was only identified from a draft genome (Keeling et al., in press) and from larval RNAseq data [17]. The specific expression of *Gr2* in larvae further suggests a role in orientation of neonates to CO_2_ detection in *D. v. virgifera*.

## Conclusion

Specific genes potentially involved in CO_2_ perception in *D. v. virgifera* have been identified and were differentially expressed among development stages and tissues. Based on expression results, *Dvv_Gr2* may be more important in host orientation of neonates. It should be noted that these results contrast those from mosquitoes and fruit flies where *Gr1* and *Gr3* have been identified as playing a more important role in CO_2_ perception. Differences in receptors between adults and larvae may explain such results. Additional studies to validate the relative importance of these genes in larval host orientation will provide insight into the relative roles for these gustatory receptors in rootworm larvae. Previous success with RNA interference in both adult and larval rootworms [36–38] should provide an effective tool for validating functions for these putative receptors through loss of function assays.

The importance of CO_2_ as an orientation cue for neonates is well documented in rootworm larvae [6] and may provide a potential mechanism to protect corn plants from rootworm damage. The identification of specific genes responsible for CO_2_ perception may provide important information for designing rootworm specific management approaches that disrupt rootworm host finding.

## Availability of supporting data

The data sets supporting the results of this article are included within the article.
